# Integrated Care Search: development and validation of a PubMed search filter for retrieving the integrated care research evidence

**DOI:** 10.1186/s12874-020-0901-y

**Published:** 2020-01-21

**Authors:** Raechel A. Damarell, Suzanne Lewis, Camilla Trenerry, Jennifer J. Tieman

**Affiliations:** 1 Flinders Filters, Flinders University, GPO Box 2100, Adelaide, South Australia Australia; 2 Library Services, Central Coast Local Health District, Gosford Hospital, PO Box 361, Gosford, NSW 2250 Australia

**Keywords:** Information retrieval, Integrated care, Literature searching, Precision, PubMed, Recall, Search filters

## Abstract

**Background:**

Integrated care is an increasingly important principle for organising healthcare. Integrated care models show promise in reducing resource wastage and service fragmentation whilst improving the accessibility, patient-centredness and quality of care for patients. Those needing reliable access to the growing research evidence base for integrated care can be frustrated by search challenges reflective of the topic’s complexity. The aim of this study is to report the empirical development and validation of two search filters for rapid and effective retrieval of integrated care evidence in PubMed. One filter is optimised for recall and the other for precision.

**Methods:**

An Expert Advisory Group comprising international integrated care experts guided the study. A gold standard test set of citations was formed from screening *Handbook Integrated Care* chapter references for relevance. This set was divided into a Term Identification Set (20%) for determining candidate terms using frequency analysis; a Filter Development Set (40%) for testing performance of term combinations; and a Filter Validation Set (40%) reserved for confirming final filter performance. In developing the high recall filter, recall was steadily increased while maintaining precision at ≥50%. Similarly, the high precision filter sought to maximise precision while keeping recall ≥50%. For each term combination tested, an approximation of precision was obtained by reviewing the first 100 citations retrieved in Medline for relevance.

**Results:**

The gold standard set comprised 534 citations. The search filter optimised for recall (‘Broad Integrated Care Search’) achieved 86.0–88.3% recall with corresponding low precision (47–53%). The search filter optimised for precise searching (‘Narrow Integrated Care Search’) demonstrated precision of 73–95% with recall reduced to between 55.9 and 59.8%. These filters are now available as one-click URL hyperlinks in the website of International Foundation for Integrated Care.

**Conclusions:**

The Broad and Narrow Integrated Care Search filters provide potential users, such as policy makers and researchers, seamless, reliable and ongoing access to integrated care evidence for decision making. These filters were developed according to a rigorous and transparent methodology designed to circumvent the challenges of information retrieval posed by this complex, multifaceted topic.

## Background

Integrated care as an organising principle of healthcare delivery is of interest to policymakers worldwide [1]. Its appeal lies in its patient-centred approach to addressing pressing concerns around rising health care costs, service fragmentation, lack of coordination across health sectors, and the burgeoning challenges presented by chronic disease, multimorbidity, and ageing populations [2]. There is no universal approach to ‘doing’ integrated care. A range of initiatives have been developed internationally but these have been implemented to meet specific local, jurisdictional, or national contexts and priorities [3]. Consequently, a multiplicity of integrated care models and approaches has given rise to an array of overlapping concepts and definitions for integrated care, all attempting to capture its complex facets, principles, mechanisms, and values [4]. This lack of a standardised, commonly understood conceptual language has arguably hindered efforts to promote common practices [5] and to develop evaluative methods capable of facilitating meaningful comparison between programs operating in dissimilar contexts [6]. As Amelung et al. state, ‘(s)uccessful integrated care programs are often a mosaic of ideas and concepts from a variety of settings that are intelligently woven together.’^3^ Notwithstanding these complexities, integration stands as an essential driver of health care reform and its growing evidence base is vital for informing policy and service design. Stakeholders therefore require convenient, reliable access to the international integrated care research to draw on current best practices.

### Challenges to finding integrated care evidence

Despite an imperative for evidence-informed integrated care policy making and system design, finding current, high quality research evidence on integrated care initiatives is challenging [7]. An assortment of terms are often used interchangeably for the concept, for example: *managed care; coordinated care; care coordination;* and *transmural care* [8]. Similarly, searchers may need to account for the multiple *dimensions* of integrated care. Here, the various taxonomies, typologies, and frameworks available on the topic may be informative as they help distinguish between the individual dimensions and their key features [6, 9, 10]. These dimensions commonly describe the foci of integration efforts (e.g. clinical, professional, organisational) and the macro, meso, and micro levels at which they take place [10]. While many of these dimensions and their features designate crucial characteristics of integrated care, they may not be exclusively associated with it. Prime examples of this are *patient centred care* and *multidisciplinary care teams.* To not include these terms in a search strategy for integrated care risks missing relevant literature. Including them, however, means retrieving an overwhelming number of citations with a high proportion of less relevant retrievals. In other words, integrated care’s lack of well-defined conceptual boundaries and tight, exclusive terminology may make searching for topic-relevant literature a poor precision exercise at best. Previous studies using bibliometric analysis to analyse publishing patterns and indexing characteristics of the integrated care research literature have also highlighted searching difficulties due to the wide range of journals publishing integrated care content, and the variable level of indexing of some key journals [11, 12].

### Search filters

Topic search filters have proven effective tools for improving the quality of evidence retrieval within large databases, especially for complex topics [13]. These are empirically derived search strategies comprising the optimal combination of search terms, database functionality, and syntax for retrieving citations describing a common subject area within a database whilst excluding citations not on that topic. Examples of complex topics which have prompted the development of a search filter include: *knowledge translation* [14]; *primary health care* [15]; *patient and public involvement in health research* [16]; and *patient views and preferences* [17]. They are often made available to users as a search string to be copied and pasted or replicated in a database. More conveniently, some exist as one-click hyperlinks in a webpage [18].

Central to filter development is the creation of a set of citations which are both relevant to the topic of interest, and which cover the full scope of that topic. This is usually called the ‘gold standard’ set. If this set is representative of the topic, it should be possible to use it to estimate a filter’s general level of performance across a full database. This grants potential users the means of knowing in advance how the tool might be expected to perform and whether it will do so at a level adequate for their own needs.

Filter performance may be measured as ‘recall’ and ‘precision’. Recall (or ‘sensitivity’) is the proportion of relevant citations retrieved by the filter out of all relevant citations in the dataset. Precision is the proportion of relevant citations retrieved out of all citations retrieved (both relevant and irrelevant). Searchers seeking comprehensive retrieval will favour high sensitivity values, even if this means having to screen many irrelevant citations to find the few relevant (i.e. low precision). This usually characterises systematic review searches [19]. Those wishing to find some, but not necessarily all relevant citations, without having to review a large number of retrievals, will favour high precision at the expense of some sensitivity. Table 1 shows the formulae for calculating these values.

**Table 1:** Search filter performance measures

Search results	Relevant citations	Irrelevant citations
Citations retrieved by search	a	b
Citations not retrieved by search	c	d
Formulae		
Sensitivity (recall) = a/(a + c) Specificity = d/(b + d) Precision = a/(a + b) Accuracy = (a + d)/(a + b + c + d) Number Needed to Read = 1/Precision		

In 2017, the International Foundation for Integrated Care (IFIC) partnered with the Central Coast Local Health District of New South Wales Health, the University of Newcastle, and the search filter research group Flinders Filters at Flinders University, South Australia, to examine the possibility of developing an integrated care search filter for the freely available PubMed database. A bibliometric study was first conducted to gain an understanding of the existing integrated care literature and where it can be located, as well as the predominant terminology associated with it [11]. From this project, we determined a search filter was not only feasible, but highly desirable due to the unique challenges posed by the topic itself. Once developed, this filter would be made available to the international integrated care community on the IFIC webpage.

Twelve international integrated care experts were invited to form an Expert Advisory Group (EAG) to provide oversight to the project and assist with tasks at certain points in the methodology. It was also important that the project team understood the EAG’s specific information needs as members represented the eventual users of the filter. The EAG was in consensus from the outset that high search precision was preferable to high recall, yet it was still concerned with not missing too many relevant articles. The project group therefore proposed two versions of the filter:

- A ‘broader’ version with the highest level of recall achievable while keeping precision ≥50%.
- A ‘narrower’ version with the highest level of precision achievable while keeping recall ≥50%.

## Objectives

This study aimed to use an objective and experimental approach to develop and validate search filters for the sensitive and precise retrieval of integrated care literature in the PubMed database for the benefit of researchers, health administrators and planners, policy makers, and clinicians. For this purpose, we chose to operationalise the concept of integrated care by giving preference to the following integrated care definition:

… a coherent set of methods and models on the funding, administrative, organisational, service delivery and clinical levels designed to create connectivity, alignment and collaboration within and between the cure and care sectors. The goal of these methods and models is to enhance quality of care and quality of life, consumer satisfaction and system efficiency for patients with complex, long term problems cutting across multiple services, providers and settings. The result of such multi-pronged efforts to promote integration for the benefit of these special patient groups is called ‘integrated care.’ [5]

If necessary, this definition would be referred to during critical decision-making points in filter development to justify directions and resolve inclusion/exclusion disagreements.

## Methods

The search filter was first developed in the Ovid Medline database and then accurately translated for PubMed. Ovid Medline was preferred for the development stage to avoid automatic processes in PubMed that would need to be accounted for and controlled, such as Medical Subject Heading (MeSH) mapping and ‘autoexploding’. There were six phases to development: forming the gold standard set; deriving candidate search terms; filter development; filter validation; filter translation for PubMed; and determining an estimate of precision we have termed the ‘post-hoc precision estimate’.

### Phase 1. Forming the gold standard set

Based on advice from the EAG, several sources of integrated care evidence were used to create a gold standard set. These were:

- References from Handbook Integrated Care [3]
- References from grey literature sources cited in chapters 1 and 2 of *Handbook Integrated Care* [3]
- Medline citations sampled from years 2010, 2013, and 2016 using the MeSH term *Delivery of Health Care, Integrated* and dual reviewed as relevant by two EAG members. This set had been created for a related study published in 2018 [11].

To be eligible for inclusion, references had to have a bibliographic record in the Ovid Medline database and be independently reviewed as relevant by two EAG members. Gold standard citations were exported from Ovid Medline into an EndNote X8 library. Using Research Randomizer [20], each citation was then randomly assigned by its EndNote record number to one of three sets:

- A term identification set (TIS) comprising 20% of citations
- A filter development set (FDS) comprising 40% of citations
- A filter validation set (FVS) comprising 40% of citations.

### Phase 2. Deriving candidate search terms

Candidate terms for the search filter could be selected from MeSH terms and/or terms in the titles and abstracts (or ‘textwords’) of TIS citations. The order in which terms were tested for the filter depended on their frequency of occurrence in the TIS. Frequencies of MeSH terms were determined separately from the frequencies of textwords.

#### MeSH term frequencies

The frequencies of MeSH terms and their subheadings were determined using the PubMed PubReMiner open source data mining tool [21], which serves as a front end to the PubMed database. PubReminer analyses elements of PubMed search results, displaying them in frequency tables. First, the PubMed Identifiers for each citation in the TIS were extracted. These numbers were then joined together in a search string separated by the Boolean operator OR and followed by the PubMed Unique Identifier search tag [UID], e.g. 24,950,517[UID] OR 16773158[UID] OR 18843691[UID] …. This string was entered as a search in the tool and the resulting MeSH term frequency table saved for subsequent analysis.

#### Textword frequencies

Textword frequencies within the TIS citations were identified using the freely available WriteWords Word Frequency Counter [22]. First, the titles and abstracts of citations in the TIS set were extracted from EndNote and saved as a text file. This file was then copied and pasted into the WriteWords search box. The program then produced frequency lists of single terms as well as double, triple, and quadruple term phrases.

#### Determining weighted frequencies

The MeSH term and textword frequency tables produced in this way ranked terms based on their frequency both *within* as well as *across* citations. This means a term occurring multiple times in one citation only might outrank a term present across multiple citations. In literature searching, a search term need only occur once within a citation for that citation to be retrieved. The number of times it occurs *within* a single citation is therefore irrelevant. The next step was therefore to determine the frequency of term occurrence across citations—a more weighted measure of frequency. For this, the TIS was reconstructed in Ovid Medline using the same search string used in PubReMiner with the PubMed Unique Identifier (UID) tag replaced with the Medline equivalent (.ui.). All MeSH terms and subheadings with a frequency of 5 or more were then searched in Medline and combined with the TIS set using Boolean AND to determine the number of TIS citations retrieved. MeSH terms and their subheadings were tested in their exploded forms when their narrower headings were also listed in the frequency table.

Single and multi-word textwords with a frequency of 5 or more in Writewords were then tested in the TIS. Frequencies were ascertained using the .tw (textword) command suffix which searches on the title and abstract fields of a Medline record. The .mp (multi-purpose) suffix was also tested when certain textwords were well represented in high frequency MeSH terms, e.g. ‘health’. (The .mp suffix searches the subject heading field in addition to the title and abstract field.) Truncated versions of single terms were tested when variant endings of the same term were prevalent in the frequency table (e.g. health, healthcare). This process resulted in a new frequency table interlacing both MeSH terms and textwords.

### Phase 3. Filter development

Each term in this new frequency table with a frequency of 27 and above (i.e. 25% recall in TIS) was now considered a candidate for the search filter. Phase 3 tested the aggregate performance of candidate terms using a different set of citations—Filter Development Set (FDS).

#### Individual term testing in the FDS

Terms were again searched individually, and their recall established in the FDS. As recall on its own is insufficient in informing a well-balanced search filter, we also took a ‘proxy’ precision estimate for each search term by:

- capturing the first 100 citations retrieved from Ovid Medline by each term *outside* the FDS, sorted by reverse chronological publication date to avoid retrieving FDS citations, and
- screening each citation for relevance to the concept of integrated care (RD and CT).

For each term we now had a baseline set of recall and proxy precision percentages to use as a starting point for testing term combinations with the aim of steadily improving search precision while sustaining recall at a level ≥ 50%.

#### Establishing concept groups

It was clear from the FDS frequency table that the top-ranking candidate terms fell into distinct groups, each group describing a different concept. This suggested that it might not be appropriate to treat listed terms as conceptually equivalent and simply combining them using the OR operator to maximise recall. Instead, terms describing different concepts might together describe integrated care when combined using AND. These combinations might lower recall but should have a positive effect on precision. To know which group each term belonged to it was therefore necessary to trial terms in combination using both OR and AND. For this, two authors (RD and CT) independently reviewed candidate terms and sorted them into concept groups. These groupings and the terms within them were then discussed by all authors and differences in opinion resolved through consensus.

#### Combining terms within and across concept groups

Next, high frequency terms within the *same* concept group were sequentially combined with each other using first OR and then the AND Boolean operator. Recall and proxy precision were calculated for each combination. This process tested the proposition that terms within each hypothesised concept group were synonyms and could improve recall when OR’d together. Various permutations of terms from *across* concept groups were then tested using the AND operator to check the effect this had on search precision. Once baseline performance measures for these AND’d combinations were established, terms were sequentially OR’d into the search string within their own concept group while the two concept groups remained AND’d with each other. Terms that could not increase recall in the FDS, or which lowered precision on their addition, were eliminated as candidate search terms. This process continued until no further improvement could be made to precision without reducing recall and vice-versa.

#### Statistical analysis of non-retrieved FDS citations

Titles and abstracts of FDS citations that could not be retrieved by the best performing search construction were exported from EndNote as a .txt file and imported into WriteWords for further frequency analysis. This revealed remaining concepts not yet explored as relevant integrated care subdomains. A new frequency table containing these terms alone was then constructed and tested in the FDS in combination with the existing search construction (ie. AND’d) and in parallel to it (i.e. OR’d with it).

#### Creating filter variants

Using the extensive recall and proxy precision data created, two variant integrated care search filters were created—one maximising recall (the broad version) while holding precision above 50%, and another favouring high precision (narrow version) while keeping recall above 50%.

### Phase 4. Filter validation

The two final filters were validated by testing their performance in the Filter Validation Set. This process makes it possible to establish filter consistency in performance across multiple sets of citations and provides some evidence as to potential generalisability across the full Medline database.

### Phase 5. Filter translation for PubMed

Final Medline integrated care search filters were translated for PubMed by converting Ovid syntax into PubMed search tags and adjusting for PubMed’s unique search algorithm. The PubMed Unique Identifiers of the TIS, FDS and FVS were first combined into one search string and run in PubMed to recreate the full gold standard set in this database. The two PubMed translations were then run in PubMed on their own and combined (AND’d) with the full gold standard set to establish recall.

To check equivalence with the original Medline search filter, this process was repeated in the Medline database using the fully reconstructed gold standard and the two Medline search filters. Retrieval in both databases was then compared for equivalence in terms of overall recall. It was also important to check, in the circumstance that the PubMed versions retrieved the exact same number of citations from the gold standard as the Medline version, if these were actually the same citations.

### Phase 6. Post hoc precision estimate

Search filter precision was put to more robust testing by asking EAG members to each review for relevance a set of 100 citations retrieved by one of several versions of the filter in the PubMed database, outside of any gold standard subsets. Fifteen sets of 100 citations each were created 9–11 October 2017 and each set was reviewed by one EAG member (i.e. no dual review). The search filters were used in three ways to produce the sets for review.

1. Five sets were retrieved using the broad filter with each set comprising citations from a different year (2012 to 2016). We chose this year range as it covers the most recent publications on the topic, with the exception of 2017/2018 citations. These years were not included in case of a MeSH indexing backlog. Such a backlog would potentially bias findings by forcing a comparison between sets of earlier, MeSH-indexed citations and more recent, largely non-indexed ones.
2. Five sets were retrieved using the narrow filter with each set comprising citations from a different year (2012 to 2016).
3. Five sets used the broad filter in combination with search terms describing a specific domain of interest to integrated care (*community health care, mental health care, aged care, rural health,* and *acute care*). Search results were sorted using PubMed’s ‘Best Match’ function before being downloaded for review. This was done to check the effect on precision when different concepts were combined with ‘integrated care’. It also reflects the way the search filter is expected to be used once publicly available.

## Results

### Phase 1. Forming the gold standard set

**Fig. 1:**
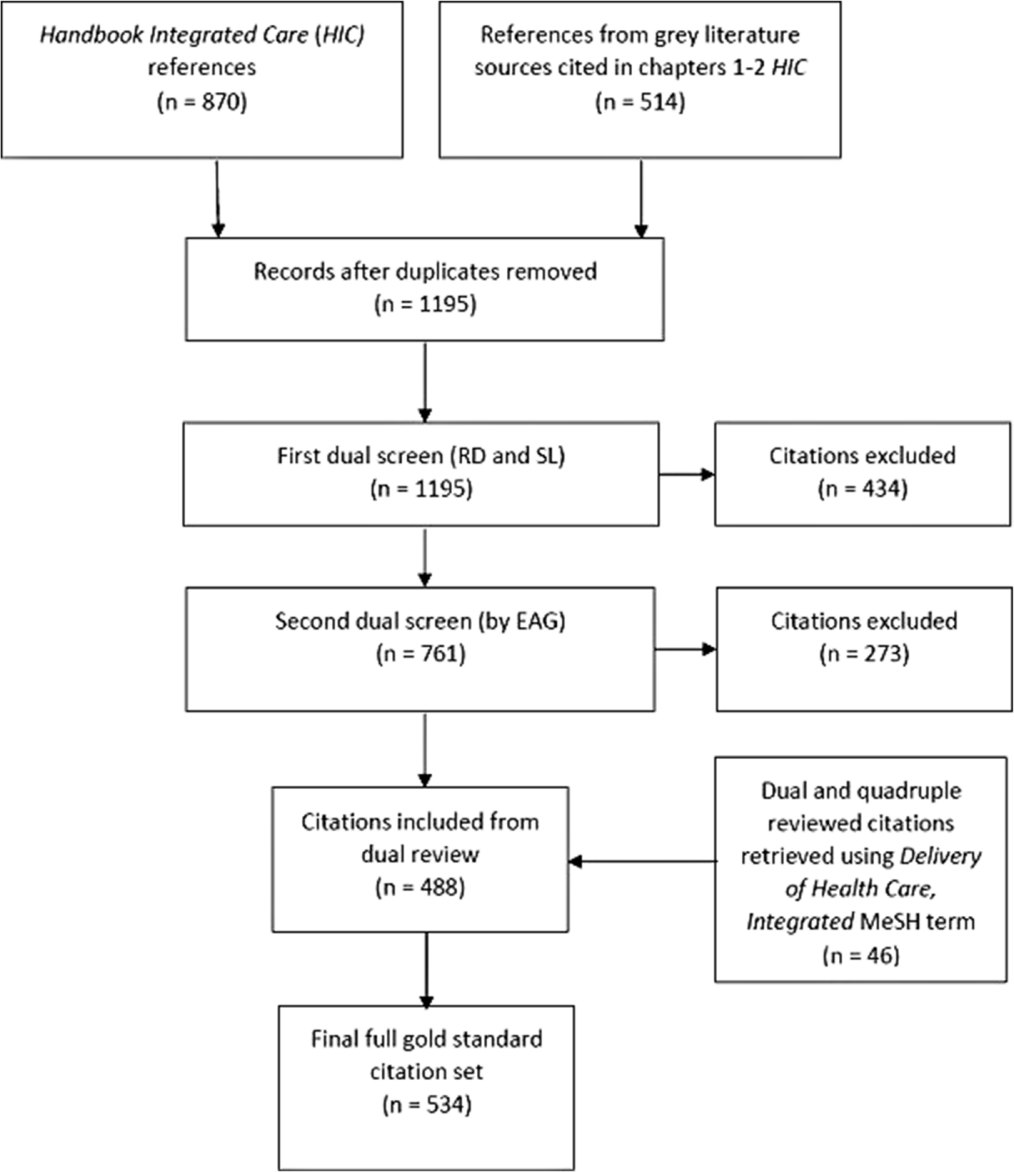
Formation of the gold standard set

The process of forming the gold standard set from three different sources is shown as Fig. 1.

#### Characteristics of the gold standard set

**Fig. 2:**
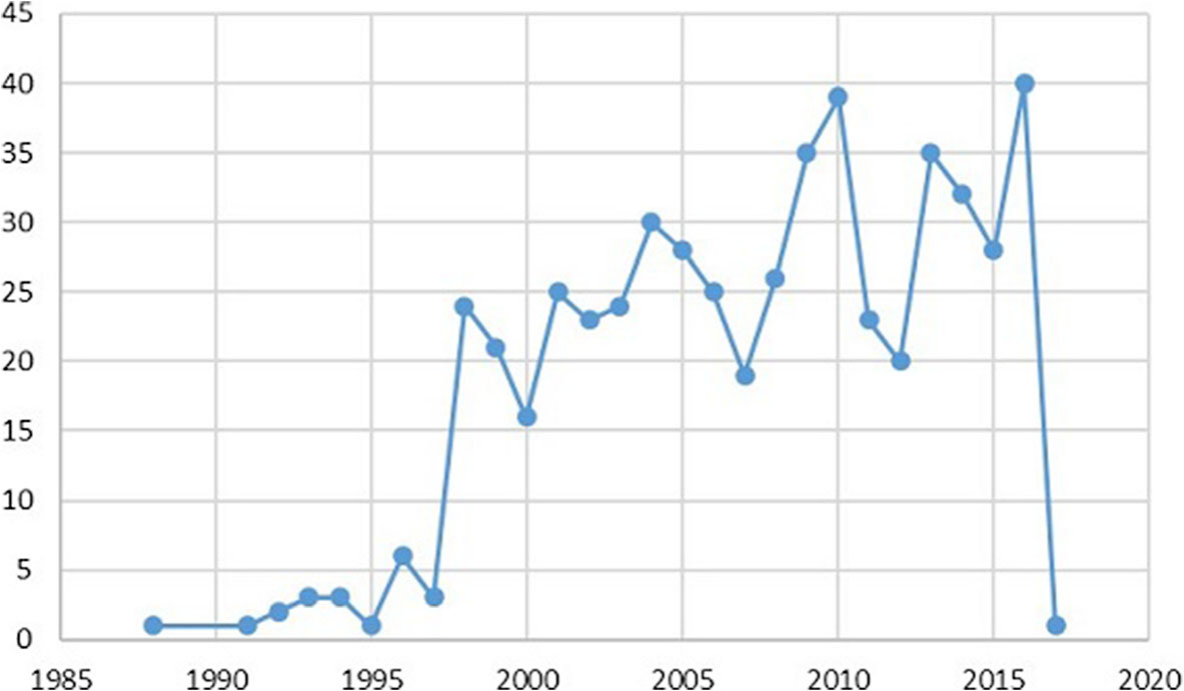
Gold standard set date coverage and year frequencies

The full gold standard set comprised *n* = 534 citations from 226 unique journal titles and spanning the years 1988 to 2017. The spread of citations across this year range is shown in Fig. 2.

The top 10 journals represented in the gold standard set are shown as Table 2.

**Table 2:** The ten highest frequency gold standard set journal titles

International Journal of Integrated Care	45
Health Affairs (Millwood)	22
BMJ	16
BMC Health Services Research	13
Cochrane Database of Systematic Reviews	13
Health Policy	12
Health & Social Care in the Community	11
American Journal of Managed Care	9
HealthcarePapers	9
Journal of the American Geriatrics Society	9

The gold standard set was split into three subsets with the following proportions of citations:

- Term Identification Set (TIS) *n* = 107 (20%)
- Filter Development Set (FDS) *n* = 213 (40%)
- Filter Validation Set (FVS) *n* = 214 (40%)

### Phase 2. Deriving candidate search terms

The MeSH and textword terms capable of retrieving the highest number of unique citations from the TIS (≥ 25%) are shown in Table 3.

**Table 3:** Highest frequency MeSH terms and textwords in the TIS

Terms	Unique citations retrieved from TIS (total *n* = 107)	% citations retrieved from TIS
MeSH		
Organization & administration.xs.	88	82.3
Delivery of health care, Integrated/	55	51.4
Economics.fs.	31	29.0
Therapy.xs.	30	28.0
Textwords/phrases		
Health*.mp.	104	97.2
Health.mp.	102	95.3
Care.mp.	102	95.3
Care.tw.	94	87.9
Health*.tw.	85	79.4
Health care.mp.	81	75.7
Integrat*.mp.	80	74.8
Integrat*.tw.	77	72.0
Health.tw.	75	70.1
Integrated.mp.	74	69.2
Services.mp.	68	63.6
Delivery.mp.	67	62.6
Integrated.tw.	58	54.2
Support.mp.	58	54.2
Patient.mp.	54	50.5
Services.tw.	49	45.8
Systems.mp.	43	40.2
Management.mp.	40	37.4
Integration.mp.	39	36.5
Organizational.mp.	39	36.5
Systems.tw.	37	34.6
Community.mp.	36	33.7
Data.mp.	36	33.7
Model.mp.	35	32.7
Practice.mp.	35	32.7
Organizational.mp.	35	32.7
Quality.mp.	34	31.8
Health care.tw.	34	31.8
Service.mp.	33	30.8
Patient.tw.	33	30.8
Community.tw.	33	30.8
Models.mp.	32	30.0
Management.tw.	32	30.0
Healthcare.tw.	32	30.0
Delivery.tw.	31	29.0
System.mp.	30	28.0
Service.tw.	30	28.0
Data.tw.	30	28.0
Hospital.mp.	30	28.0
Primary care.mp.	30	28.0
Primary care.tw.	29	27.1
Clinical.mp.	29	27.1
Hospital.tw.	28	26.2
Disease.mp.	28	26.2
Design.mp.	27	25.2
^±^Coordinat*.mp.	21	19.6

.mp = search on title, abstract, keywords, and subject headings

.tw = search on title and abstract

.xs = search on exploded free-floating subheading

.fs = search on free-floating subheading (not exploded)

/ = Medical Subject Heading (MeSH) search

? allows for single letter variants within a word (here: *organisational* OR *organizational*)

^±^Coordinat* was chosen from lower down the TIS-derived frequency list as a possible equivalent term for ‘integrated’

### Phase 3. Filter development

#### Individual term testing in the FDS

The highest frequency textwords from the TIS were again searched in the FDS to determine their recall. Their corresponding precision was also estimated in Medline outside of the FDS. Although recall for some terms was high (e.g. care.mp at 98.1%), precision proved very low (see Table 4). The term with the most face validity—integrated care—had low recall in the FDS (43/213; 20.2%) so it was not considered a candidate term at this stage. Similarly, the most relevant MeSH term, “Delivery of Health Care, Integrated”, had low recall, retrieving only 95/213 citations, or 44.6% of the FDS.

**Table 4:** FDS recall and PubMed precision of highest-ranking candidate terms

Searches	Recall in FDS (*n* = 213)		% Precision in PubMed (Total *n* = 100)
	n	%	
Integrat*.mp.	159	74.7	8
Integrat*.tw.	152	71.4	8
Integrated.mp.	143	67.1	8
Integrated.tw.	118	55.4	8
Coordinat*	46	21.6	8
Care.mp.	209	98.1	0
Care.tw.	180	84.5	0
Health*.mp.	199	93.4	0
(Health OR healthcare).mp	199	93.4	0
Health.mp.	197	92.5	0
Health.tw	143	67.1	0

#### Establishing concept groups

**Fig. 3:**
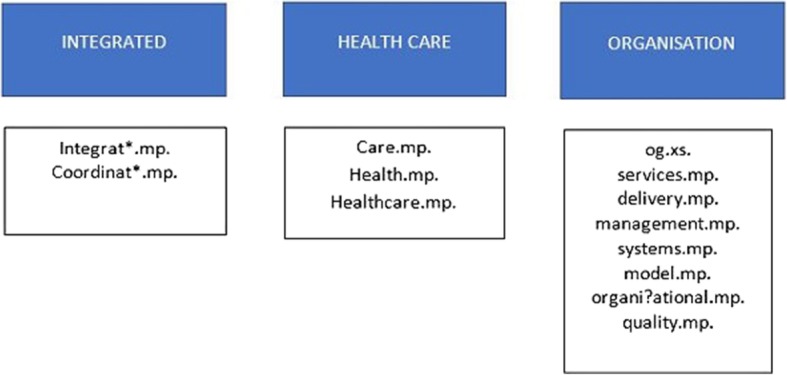
Concepts groups and their relevant terms

Concept groupings of high frequency candidate terms were hypothesised as: [1] integrated [2] health care [3] organisation and administration. These groups and the terms that fall under each are shown in Fig. 3.

In og.xs., the ‘og’ is the abbreviated form of Medline subheading ‘organization & administration’. In its exploded form (indicated by .xs) it also includes a search on the related subheadings: economics; legislation & jurisprudence; manpower; standards; supply & distribution; trends; and utilization.

#### Combining terms within and across concept groups

The FDS was then used to test the best performing combinations of terms from the first two concept groups, ‘integrated’ and ‘health/care.’ To determine the most meaningful way to combine them, each term was tested with the other terms in its own group and then with terms in the other group. However, when the high frequency terms were tested *within their concept groups*, proxy precision remained very low, often at 0%, for both the OR and the AND Boolean operators while recall stayed at an acceptable level.

As expected, the OR operator outperformed the AND operator at maintaining recall with no clear effect on precision. Table 5 shows the initial results of this process using the first two concept groups only.

**Table 5:** Sequential testing of terms within two concept groups in the FDS

Searches	Recall in FDS (*n* = 213)		Proxy precision in Medline (Total *n* = 100)
	n	%	%
OR’d combinations (*within* concept group)			
Health.mp. OR healthcare.mp.	199	93.4	0
Care.mp. OR health.mp.	211	99.1	0
Care.mp. OR health*.mp	211	99.1	0
^#^(Care.mp. OR health OR healthcare).mp.	211	99.1	0
Care.tw. OR health.tw.	194	91.1	0
Integrat*.mp OR coordinat*.mp.	166	77.9	3
AND’d combinations (*within* concept group)			
Health.mp AND healthcare.mp	43	20.2	0
Care.mp. AND health.mp.	195	91.6	0
Care.mp. AND health*.mp	197	92.5	0
^#^Care.mp. AND (health.mp. OR healthcare.mp)	197	92.5	0
Integrat*.mp AND coordinat*.mp.	39	18.3	0

At this stage, it was too soon to decide between the OR and the AND combinations involving ‘care’ and variants on ‘health’ (indicated by preceding symbol #) as both combinations achieved recall above 90% with similar poor precision. However, the truncated form ‘health*’ was here dropped as an option based on two observations:

1. Once the filter is translated for PubMed, retrieval on ‘health*’ would be capped at the first 600 word ending variants, which may reduce recall equivalency between the Ovid Medline and PubMed search filter versions.
2. Health* has the same level of recall as ‘health OR healthcare’ when both versions were combined with ‘care.mp.’ (197/213; 92.5%).

When the two concept groups, ‘integrated’ and ‘health/care’, were combined with each other using AND, a significant increase in proxy precision occurred alongside a drop in recall. This effect continued as more terms were successively added to the ‘health/care group until precision reached 56%. Table 6 shows the progressive improvement in precision as successive ‘within group’ terms were added to the basic two concept search.

**Table 6:** Sequential testing of combined concepts (‘integrated’ and ‘health/care’) in the FDS

Searches	Recall in FDS (*n* = 213)		Proxy precision in Medline (*n* = 100)
	n	%	%
(Integrat* OR coordinat*).mp AND health.mp.	156	73.2	28
(Integrat* OR coordinat*).mp AND healthcare.mp	36	16.9	35
(Integrat* OR coordinat*).mp AND health*.mp	157	73.7	25
(Integrat* OR coordinat*).mp. AND care.mp.	163	76.5	40
OR’d combinations			
(Integrat* OR coordinat*).mp AND (health OR healthcare).mp	157	73.7	33
(Integrat* OR coordinat*).mp. AND (care OR health OR healthcare).mp.	165	77.5	30
AND’d combinations			
^#^(Integrat* OR coordinat*).mp. AND care.mp. AND (health OR healthcare).mp.	155	72.8	56
(Integrat* OR coordinat*).mp. AND ((care AND health) OR healthcare).mp.	155	72.8	49

The best candidate combination was determined to be the search indicated by the #. This is: (Integrat* OR coordinat*).mp. AND care.mp. AND (health OR healthcare).mp. This construct kept precision above 50% without significantly reducing recall.

Each of the remaining terms in the frequency table were then tested in combination with this construct in three ways:

1. Combined with the construct using AND
2. Combined with the construct using OR
3. Combined within the health/care construct using OR to test if synonymous with that concept.

Terms that reduced precision on their addition to the search construction, or which could not maintain or increase recall when precision remained steady, were eliminated from the developing search string. This included the MeSH term *Delivery of health care, Integrated* and textwords: *support, patient(s), community, data, hospital, primary care, clinical, disease,* and *design.*

The final best performing search at the end of this process was:

((Integrat* OR coordinat*) AND care AND (health OR healthcare)).mp. AND (og.xs. OR services.mp. OR delivery.mp. OR management.mp. OR systems.mp. OR model.mp. OR organi?ational.mp. OR quality.mp.)

This search string, labelled Search Component 1, has 71.8% recall (153/213) and 62% proxy precision in the FDS. The fact that it was unable to retrieve *n* = 60 (28.2%) of citations from the FDS suggested other concepts and terms closely associated with integrated care may remain unidentified in the FDS. Although these terms were not of sufficiently high frequency to be identified within the TIS recall cut-off threshold of ≥25%, they may serve as highly discriminatory search terms.

#### Statistical analysis of non-retrieved FDS citations

When the titles and abstracts of the remaining 60 FDS citations were submitted to frequency analysis using WriteWords, two high frequency terms emerged: ‘disease management.mp.’ and ‘case management.mp’. These two terms were trialled using a process parallel to the one used to build Search Component 1, i.e. by successively adding concept groups to this new concept group to steadily improve precision while keeping recall close to an acceptable baseline. Details of this are provided as Additional file 1.

Table 7 shows the final ‘disease management’ concept search (Search Component 2) and its effect on overall recall and precision when combined with Search Component 1.

**Table 7:** Search Components 1 and 2 within the FDS

Searches	Recall in FDS (*n* = 213)		Proxy precision in Medline (*n* = 100)
	n	%	%
Component 1			
((Integrat* OR coordinat*) AND care AND (health OR healthcare)).mp. AND (og.xs. OR services.mp. OR delivery.mp. OR management.mp. OR systems.mp. OR model.mp. OR organi?ational.mp. OR quality.mp.)	153	71.8	62
Component 2			
(Disease management OR Case management).mp. AND (care OR health OR healthcare).mp. AND (og.xs. OR services.mp. OR delivery.mp. OR model.mp. OR quality.mp.)	55	25.8	69
Component 1 OR component 2			
(((Integrat* OR coordinat*) AND care AND (health OR healthcare)).mp. AND (og.xs. OR services.mp. OR delivery.mp. OR management.mp. OR systems.mp. OR model.mp. OR organi?ational.mp. OR quality.mp.)) OR (((Disease management OR Case management) AND (care OR health OR healthcare)).mp. AND (og.xs. OR services.mp. OR delivery.mp. OR model.mp. OR quality.mp.))	180	84.5	63

This left 33 citations not retrieved by this search. Of these, five citations contained the low frequency textword ‘Integrated care’ and were from the *International Journal of Integrated Care (IJIC)—*a key journal title for researchers within the field of integrated care. These citations had not been retrieved for one of two reasons: [1] they did not contain any of the other search terms from Search Component 1 (e.g. care OR health/care) and [2] they were not indexed with MeSH terms or lacked an abstract. In fact, as of 5 October 2017, 26% of all IJIC citations (146/558) lacked an abstract making them only retrievable via terms in the article or journal title. Based on this information, we tested the addition of the straight phrase ‘Integrated care’ to the search construction as both a journal title keyword (.jw) and a search on title, abstract and MeSH terms (.mp.)

Integrated care.mp,jw. OR (((Integrat* OR coordinat*) AND care AND (health OR healthcare)).mp. AND (og.xs. OR services.mp. OR delivery.mp. OR management.mp. OR systems.mp. OR model.mp. OR organi?ational.mp. OR quality.mp.)) OR (((Disease management OR Case management) AND (care OR health OR healthcare)).mp. AND (og.xs. OR services.mp. OR delivery.mp. OR model.mp. OR quality.mp.))

This addition of ‘integrated care’.mp,jw to the search retrieved all five IJIC citations and increased recall to 88.3% (188/213) within the FDS—an increase of 3.8%. Although this is a slight increase, we retained the .jw search element as the journal was uniquely identified with the integrated care concept. Furthermore, the EAG agreed that comprehensive retrieval would be supported by inclusion of content from this journal. Currently no other journals are picked up by searching ‘integrated care’ across the journal title field in Medline.

**Fig. 4:**
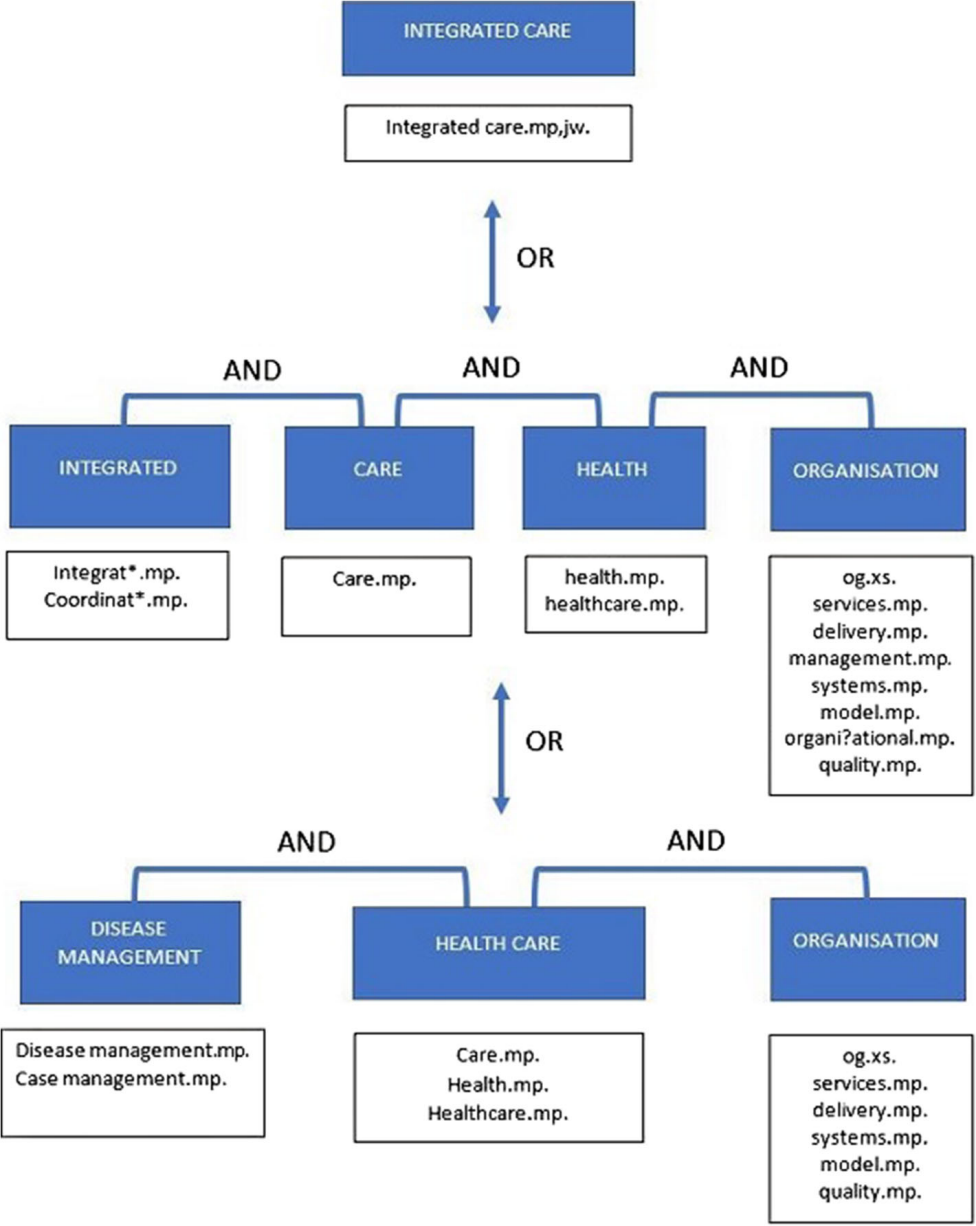
Conceptual diagram of *Broad ICS*

The final Ovid Medline search filter (above) therefore achieved 88.3% recall in the FDS (95% CI [83.3–91.9]) with a reduced final proxy precision of 53%. As this constitutes high recall with precision very close to the minimal level of acceptance, this search filter was designated *Broad Integrated Care Search* (or *Broad ICS*). The overall conceptual model of *Broad ICS* is shown in Fig. 4.

#### Creating filter variants

A narrower (or more precise) integrated care search filter was created by returning to the TIS frequency table and testing less frequent terms with high face validity for their proxy precision in the FDS. Terms with individual levels of precision ≥75% in the FDS were then systematically and successively tested in combination with each other until maximum proxy precision was reached without allowing recall to go below 50%. The combination with the best level of precision was:

*Delivery of health care, integrated/ OR Integrated care.mp,jw. OR (integrated health*.mp. AND og.xs.)

This construct included a ‘focused’ version of the MeSH term *Delivery of health care, Integrated* as indicated by the asterisk before the term. This restricts retrieval to articles deemed by an indexer to have a major focus on this concept. This version of integrated care search achieved only 55.9% recall (117/213) in the FDS (95% CI [49.2–62.4]) but a precision estimate of 95% outside of the FDS. We have designated it *Narrow Integrated Care Search* (or *Narrow ICS*)*.*

### Phase 4. Filter validation

When both versions of the filter were searched within the FVS (*n* = 214), the results were:

- *Broad ICS:* 86.0% recall, 95% CI [80.7–90.0]
- *Narrow ICS:* 59.8% recall, 95% CI [53.1–66.2]

Between the FDS and FVS, recall differed by 2.2% for the *Broad ICS* and 3.9% for the *Narrow ICS.*

### Phase 5. Filter translation for PubMed

The main differences between the Medline version and its PubMed translation is the inability to directly translate Ovid’s single character wildcard? within ‘organi?ational’ for PubMed. This meant having to spell out the different forms of the term within PubMed (i.e. organizational OR organisational). The PubMed versions of both filters are shown as Table 8.

**Table 8:** Final PubMed translations of Ovid Medline ICS search filters

	Ovid Medline version	PubMed translation
Broad ICS	Integrated care.mp,jw. OR (((Integrat* OR coordinat*) and care and (health OR healthcare)).mp. and (og.xs. OR services.mp. OR delivery.mp. OR management.mp. OR systems.mp. OR model.mp. OR organi?ational.mp. OR quality.mp.)) OR (((Disease management OR Case management) and (care OR health OR healthcare)).mp. and (og.xs. OR services.mp. OR delivery.mp. OR model.mp. OR quality.mp.))	Integrated care[tw] OR integrated care[ta] OR (((Integrat*[tw] OR coordinat*[tw]) AND care[tw] AND (health[tw] OR healthcare[tw])) AND (og[sh] OR services[tw] OR delivery[tw] OR management[tw] OR systems[tw] OR model[tw] OR organisational[tw] OR organizational[tw] OR quality[tw])) OR ((Disease management[tw] OR Case management[tw]) AND (care[tw] OR health[tw] OR healthcare[tw]) AND (og[sh] OR services[tw] OR delivery[tw] OR model[tw] OR quality[tw]))
Narrow ICS	*Delivery of health care, integrated/ OR Integrated care.mp,jw. OR (integrated health*.mp. and og.xs.)	(Delivery of health care, integrated[majr:noexp] OR Integrated care[tw] OR Integrated care[ta] OR (integrated health*[tw] AND og[sh]))

*Narrow ICS* (PubMed version) retrieved 312/534 (58.4%) of the fully reconstructed gold standard set in PubMed and *Narrow ICS* (Medline) retrieved the same proportion of the gold standard within Ovid Medline. Similarly, the two versions of *Broad ICS* retrieved 467/534 (87.5%) of the gold standard set in their respective databases. An examination of the set of citations not retrieved by each version revealed them to be identical, meaning the PubMed broad and narrow ICS versions have both quantitative and qualitative equivalence with their Medline counterparts.

### Phase 6. Post hoc precision estimate

The results of the post hoc precision analysis of retrieved citations from PubMed are shown in Table 9. All final performances for both filters are provided in Table 10.

**Table 9:** Post hoc precision estimates for three variant sets of retrievals across PubMed

	Broad ICS 2012–2016 sets (%)	Broad ICS + topic search terms, sorted by Best Match (%)	Narrow ICS 2012–2016 sets (%)
Reviewer 1	37	83 (Community health)	62
Reviewer 2	55	52 (Mental health)	68
Reviewer 3	40	70 (Aged care)	83
Reviewer 4	48	78 (Rural health)	71
Reviewer 5	57	71 (Acute care)	81
Average post hoc precision (%) across five variant sets (CI)	47% 95% CI [43 to 52%]	71% 95% CI [67 to 75%]	73% 95% CI [69 to 77%]

**Table 10:** Final performance of filters

Search filter version	Recall in FDS (%) 95% CI	Recall in FVS (%) 95% CI	Post hoc precision (%) (Single set of *n* = 100 citations)	Averaged post hoc precision (%) (Five sets of *n* = 100 citations)
Broad ICS	88.3(83.3–91.9)	86.0 (80.7–90.0)	53.0	47.0
Narrow ICS	55.9 (49.2–62.4)	59.8 (53.1–66.2)	95.0	73.0

## Discussion

This study reports the development and validation of the first available search filters for locating evidence on integrated care initiatives in the open access PubMed database. By following a well-established, systematic, and objective methodology, we created two filters capable of claiming a known level of performance in this database. The narrow ICS filter is optimised for more targeted, practical searching. It has a precision rate maximised between 73 and 95% but with correspondingly low levels of recall (56–60%). The broader ICS filter is optimised to retrieve a higher proportion of all relevant citations, although this means also retrieving many irrelevant ones. While its recall could be maximised to 86–88%, precision reduced to between 47 and 53%.

This study confirms the challenges of searching for integrated care literature previously reported [11]. Firstly, it proved difficult to find a suitably broad-ranging set of resources from which to derive an adequately sized gold standard set of citations. To date there remains little consensus, or even debate, around the minimum number of citations required to create an adequately powered gold standard set. One study posits the figure of 100 citations [23]; but this number relates to the development of methodological, rather than topic search filters. For a topic as multidimensional as integrated care we believed a much larger number of citations was required to cover the depth and scope of the topic. However, systematic reviews proved too narrowly focused on singular aspects of integrated care such as ‘integrated mental health services’ or ‘multidisciplinary clinics.’ We were also not confident that enough integrated care systematic reviews existed for their included citations to form an adequately sized gold standard.

The edited textbook *Handbook Integrated Care* [3]*,* recommended by the EAG, eventually proved a convenient and current source of articles as its chapters cover a range of topics across the subject, from definitions of integrated care to patient preferences, disease management, governance, culture, values and healthcare workforce. This text also allowed us to trial a different method for developing the gold standard set as we are unaware of any filters built using monograph references. Once again, however, many of the textbook chapter references were for grey literature reports, or articles in the *International Journal of Integrated Care* which, at the time, lacked MeSH indexing and often an abstract to aid retrieval. Furthermore, many of the chapter references proved of peripheral relevance to the central topic. It was therefore necessary to screen each textbook citation for eligibility. This was done by two pairs of reviewers. Authors RD and SL first removed clearly irrelevant references before two EAG experts independently screened the remaining set. These experts were not required to resolve any differences in opinion through consensus. This means the final set ended up comprising citations that had been voted as relevant by four different reviewers. This stringent eligibility process sharply reduced the number of citations eligible for the gold standard set from 1195 to 488, highlighting the multifaceted nature of integrated care and the small proportion of studies on the topic ‘universally’ recognised as relevant. This same process occurred in a preceding, related study [11] when 300 citations retrieved by the *Delivery of Health Care, Integrated* MeSH term were screened by two to four reviewers. Of the 300 citations reviewed, only 46 were deemed relevant by all reviewing experts. These 46 were added to our gold standard set to increase its size (see Fig. 1). This rigorous standard for determining inclusion should have resulted in a test set of core—rather than peripheral—relevance, perhaps in turn biasing filter performance towards optimal precision rather than sensitivity. For this reason, users should be aware that retrieval on integrated care from specialty journal titles might be impaired if those journals use less frequent and more discipline-specific terms to describe the concept. It will be important to monitor the utility of the search filters over time and make refinements as the scope of integrated care across health research, practice, and policy becomes clearer.

The heterogeneity of concepts and terms for integrated care also challenged search development. The two most likely search strategy candidates proved to have unacceptably low recall. These were the textword ‘integrated care’ (28.5% recall) and the MeSH term ‘Delivery of Health Care, Integrated’ (44.6% recall). Retrieval was clearly confounded by the large number of ‘integrated care’ term variants such as ‘integrated end of life care,’ ‘integrated primary health care,’ ‘health systems integration’, ‘integrated geriatric care,’ and ‘integrated model of care.’ This problem of having additional words intervening between terms ‘integrated’ and ‘care’ could have been resolved more elegantly in the Medline database where an adjacency operator is available. This command facilitates retrieval where two terms occur within a maximal, predetermined number of words from each other. As PubMed does not have this functionality, we had to resort to the less precise and overly sensitive AND operator to identify the many variants on ‘integrated care’. Creating the PubMed version was, however, essential to allow engagement with an open access database and enable hyperlinked search deployment through a web interface.

The integrated care search filters are somewhat unique in utilising the Boolean operator AND in their construction, in addition to the usual OR operator. Other topic filters employing AND include those on quality improvement [24], patient safety [25], Australian Indigenous health [18], and emerging technologies [26]. These might all be considered complex, multi-concept topics. Most search filters aim to maximise search sensitivity/recall by employing a variety of synonyms combined by OR, as this operator broadens the search and increases recall. This approach works well when the topic is conceptually discrete, for example heart failure [27], the United Kingdom [28], or paramedics [29]. However, integrated care might be best understood as a constellation of smaller, independent yet overlapping concepts, rather than a single overarching concept. This was evident from the large number of textwords and several MeSH subheadings that appeared near the top of the term frequency rankings relating to the concept of healthcare organisation and delivery. These included subheadings ‘economics’ and ‘organization & administration’ and textwords ‘services’, ‘delivery’, ‘management’, ‘organisational,’ ‘systems,’ and ‘quality’. This revealed that our gold standard set of citations did not merely describe a form of care designated ‘integrated;’ they also conveyed some aspect of its organisation and delivery. In the same way, ‘disease management’ on its own was inadequate as a search term. It also needed to be combined using AND to terms descriptive of its organisation and administration.

Extensive testing of both AND and OR combinations was necessary but resource intensive. Testing terms singularly and in combination both within the FDS and outside of it required hours of work across many weeks. Many of the tasks required seem well suited to automated methods. These methods should be developed as a matter of priority for search filter development to be considered feasible and sustainable where highly complex topics are concerned. Arguably, it is the very complexity of a topic that drives the value and utility of the search filter. This may have especial importance given the difficulties that many clinicians have in effectively searching for relevant literature [30].

The Integrated Care Search filters have now been implemented in the website of the International Foundation for Integrated Care [31] where they can be used simply by clicking on a hyperlink. Here users can select from the Broad or Narrow ICS and then couple it with a more focused topic of their choice. Topics have been organised by setting (e.g. aged care, palliative care), specific populations (e.g. children, adolescents, rural populations), geographic regions, and even specific facets of integrated care such as person-centred care or governance and accountability.

### Strengths and limitations

This study benefited from the close involvement of an international group of integrated care subject experts (the EAG). This group assisted the project from its conception and the operationalisation of a definition, right through to an evaluation of the final product. In doing so it helped improve the potential usefulness of the end product to a broad range of stakeholders. Understanding the EAG’s needs also led us to create two filters with different levels of recall and precision, both meeting our predetermined targets for recall and precision.

The study sought to adhere to an objective methodology throughout, explicitly testing each variation of the filter, using AND as well as OR to combine terms when usual practice might have suggested one operator over the other. We believe such rigour and transparency in process has resulted in a highly defensible product. Some decisions were, however, made arbitrarily which may have influenced the final filter and its performance in unforeseen ways. For example, we specified that terms had to have a retrieval rate of 25% in the TIS to be considered candidates for testing in the FDS. This threshold was chosen subjectively and pragmatically after viewing the extensive list of terms identified by frequency analysis and in consideration of the time it would have taken to test them all individually. By setting the level so high, we may have missed some highly discriminatory terms lower in the rankings which might have increased both recall and precision. We also set an arbitrary threshold of 50% for both recall and precision. Future studies may quantify the minimum levels deemed satisfactory to end-users, depending on their information needs.

Precision estimation for each search variation was a crude measure but a necessary one. Determining the effect of term decisions on precision is made possible when the gold standard is created using the traditional, but resource intensive, hand search method. This involves dual screening all articles within a pre-determined range of journal titles to create a closed system of both relevant and irrelevant citations where the relevance of each item is known. As our gold standard set comprised only relevant citations, measuring the number of irrelevant citations brought in by each modification to the search had to be done some other way. Our process might be improved by having an automated way to select 100 random citations from right across all years of the database rather than taking the first 100 retrieved.

It seemed reasonable to use multiple chapters of the *Handbook Integrated Care* to form the majority portion of the gold standard as this was a multi-author, edited work. However, a check of contributor affiliations revealed a significant proportion of European authors across the 37 chapters. We have no way of knowing if these authors were invited to contribute based on a common understanding of integrated care that might not be generalisable to non-European parts of the world. Furthermore, unlike clinical practice guidelines and systematic reviews (commonly used gold standard sources), a textbook of this type need not document how its references were identified and selected for inclusion. Chapter references were most likely ‘cherrypicked’ to support the views of the author, rather than systematically sought using comprehensive, objective, or consensus methods. Taken together, this means our gold standard set is most likely biased in subtle ways. However, in reviewing the characteristics of the gold standard set any bias seems unlikely to have compromised the performance of the search filters. The literature represented covers a wide range of years and journal titles. Top 10 journals range in foci from intervention effectiveness (*Cochrane Database of Systematic Reviews*), health policy (*Health Policy*), healthcare research (*BMC Health Services Research*) to general biomedical (*BMJ*). Several top titles originate in the United States.

## Conclusions

Policy makers, researchers and clinicians need quick and efficient access to integrated care evidence to identify integrated models of care with potential to reduce costs and increase the quality and person-centredness of services. Searching for integrated care evidence is, however, challenging due to the large number of overlapping concepts that together define the topic and the heterogenous terminology used to describe it. We developed, tested, and validated the performances of two search filters for retrieving integrated care evidence from the open access PubMed database. Users select the one they need based on their purpose for searching. Broad ICS is optimised to retrieve as much of the relevant integrated care literature as possible without allowing retrieval precision to fall far below the 50% mark. This ensures that around half of the citations retrieved should be relevant. Narrow ICS, however, ensures a higher proportion of relevant citations are retrieved at the risk of not identifying as much as half of all relevant citations in the database. These search filters are now available for one-click searching on the website of the International Foundation for Integrated Care [31].

## Supplementary information

**Additional file 1.** Detailed development of Search Component 2.

## Data Availability

The datasets used and/or analysed during the current study are available from the corresponding author on reasonable request.

## Abbreviations

EAG: Expert Advisory Group
FDS: Filter Development Set
Fs: Free floating subheading (Medline)
FVS: Filter Validation Set
HIC: Handbook Integrated Care
ICS: Integrated Care Search
IFIC: International Foundation for Integrated Care
Jw: Journal title keyword (Medline)
MeSH: Medical Subject Heading (Medline and PubMed)
Mp: Multi-purpose search (Medline title, abstract and MeSH term search)
Og: Organisation and Administration subheading (Medline)
TIS: Term Identification Set
Tw: Textword search (Medline title and abstract search)
UID: Unique Identifier (PubMed)
Xs: Exploded free floating subheading (Medline)

## References

[1] Shaw S, Rosen R, Rumbold B (2011). What is integrated care? Research report.

[2] Ham C, Walsh N (2013). Making integrated care happen at scale and pace: lessons from experience.

[3] Amelung V, Stein V, Goodwin N, Balicer R, Nolte E, Suter E, Amelung V, Stein V, Goodwin N, Balicer R, Nolte E, Suter E (2017). Preface. Handbook integrated care.

[4] Armitage GD, Suter E, Oelke ND, Adair CE (2009). Health systems integration: state of the evidence. Int J Integr Care.

[5] Kodner DL, Spreeuwenberg C (2002). Integrated care: meaning, logic, applications, and implications--a discussion paper. Int J Integr Care.

[6] Busetto L, Luijkx K, Vrijhoef HJM (2017). Advancing integrated care and its evaluation by means of a universal typology. Int J Care Coord.

[7] Reed J, Childs S, Cook G, Hall A, McCormack B (2007). Integrated care for older people: methodological issues in conducting a systematic literature review. Worldviews Evid-Based Nurs.

[8] Kodner DL (2009). All together now: a conceptual exploration of integrated care. Healthcare Q (Toronto, Ont).

[9] Calciolari S, González L, Goodwin N, Stein V. The project Integrate framework: EU Project INTEGRATE; 2016. http://www.projectintegrate.eu.com/wp-content/uploads/2017/04/The-Project-Integrate-Framework-TOP.pdf. Accessed 3 Oct 2019

[10] Valentijn PP, Boesveld IC, van der Klauw DM, Ruwaard D, Struijs JN, Molema JJ (2015). Towards a taxonomy for integrated care: a mixed-methods study. Int J Integr Care.

[11] Lewis S, Damarell RA, Tieman JJ, Trenerry C (2018). Finding the integrated care evidence base in PubMed and beyond: a bibliometric study of the challenges. Int J Integr Care.

[12] Sun X, Tang W, Ye T, Zhang Y, Wen B, Zhang L (2014). Integrated care: a comprehensive bibliometric analysis and literature review. Int J Integr Care.

[13] Damarell RA, May N, Hammond S, Sladek RM, Tieman JJ (2019). Topic search filters: a systematic scoping review. Health Inf Libr J.

[14] McKibbon KA, Lokker C, Wilczynski NL, Haynes RB, Ciliska D, Dobbins M (2012). Search filters can find some but not all knowledge translation articles in MEDLINE: an analytic survey. J Clin Epidemiol.

[15] Brown L, Carne A, Bywood P, McIntyre E, Damarell R, Lawrence M (2014). Facilitating access to evidence: primary health care search filter. Health Inf Libr J.

[16] Rogers M, Bethel A, Boddy K (2017). Development and testing of a medline search filter for identifying patient and public involvement in health research. Health Inf Libr J.

[17] Selva A, Sola I, Zhang Y, Pardo-Hernandez H, Haynes RB, Martinez Garcia L (2017). Development and use of a content search strategy for retrieving studies on patients’ views and preferences. Health Qual Life Outcomes.

[18] Tieman JJ, Lawrence MA, Damarell RA, Sladek RM, Nikolof A (2014). LIt.Search: fast tracking access to aboriginal and Torres Strait islander health literature. Aust Health Rev.

[19] Sampson M, Zhang L, Morrison A, Barrowman NJ, Clifford TJ, Platt RW (2006). An alternative to the hand searching gold standard: validating methodological search filters using relative recall. BMC Med Res Methodol.

[20] Urbaniak GC, Plous S. Research Randomizer [Computer software]. Version 4.0. 2013. http://www.randomizer.org/. Accessed 3 Oct 2019.

[21] Koster J. PubMed PubReMiner [Computer program]. Version 1.31. 2014. https://hgserver2.amc.nl/cgi-bin/miner/miner2.cgi. Accessed 3 Oct 2019.

[22] WriteWords. Word frequency counter. 2002–2019. http://www.writewords.org.uk/word_count.asp. Accessed 3 Oct 2019.

[23] Yao X, Wilczynski NL, Walter SD, Haynes RB (2008). Sample size determination for bibliographic retrieval studies. BMC Med Inform Decis Mak.

[24] Hempel S, Rubenstein LV, Shanman RM, Foy R, Golder S, Danz M (2011). Identifying quality improvement intervention publications--a comparison of electronic search strategies. Implement Sci.

[25] Tanon AA, Champagne F, Contandriopoulos AP, Pomey MP, Vadeboncoeur A, Nguyen H (2010). Patient safety and systematic reviews: finding papers indexed in Medline, Embase and CINAHL. Qual Saf Health Care.

[26] Varela-Lema L, Punal-Rioboo J, Accion BC, Ruano-Ravina A, Garcia ML (2012). Making processes reliable: a validated PubMed search strategy for identifying new or emerging technologies. Int J Technol Assess Health Care.

[27] Damarell RA, Tieman J, Sladek RM, Davidson PM (2011). Development of a heart failure filter for Medline: an objective approach using evidence-based clinical practice guidelines as an alternative to hand searching. BMC Med Res Methodol.

[28] Ayiku L, Levay P, Hudson T, Craven J, Barrett E, Finnegan A (2017). The Medline UK filter: development and validation of a geographic search filter to retrieve research about the UK from OVID medline. Health Inf Libr J.

[29] Olaussen A, Semple W, Oteir A, Todd P, Williams B (2017). Paramedic literature search filters: optimised for clinicians and academics. BMC Med Inform Decis Making.

[30] Damarell RA, Tieman JJ (2016). Searching PubMed for a broad subject area: how effective are palliative care clinicians in finding the evidence in their field?. Health Inf Libr J.

[31] International Foundation for Integrated Care (2019). Integrated Care Search.

