# Supplementary material for: Integrated Care Search: development and validation of a PubMed search filter for retrieving the integrated care research evidence
Source: BMC Med Res Methodol. 2020 Jan 21;20:12. doi: 10.1186/s12874-020-0901-y (PMC6971984; doi:10.1186/s12874-020-0901-y)
Supplement: Supplementary file 1 — Additional file 1. Detailed development of Search Component 2. [file 12874_2020_901_MOESM1_ESM.docx]

## Development of search component two: ‘disease/case management’

Table 11. Testing of disease management/case management terms

| **Searches** | **Recall in FDS**  **(n=213)** | | **% Precision in PubMed (Total n=100)** |
| --- | --- | --- | --- |
|  | **n** | **%** |  |
| Disease management.mp | 30 | 14.08 | 23 |
| (Disease management or Case management).mp. | 57 | 26.76 | 29 |
| (Disease management or Case management).mp. and care.mp. | 55 | 25.82 | 57 |
| **OR combinations** | | | |
| (Disease management or Case management).mp. and (care OR health or healthcare).mp. | 56 | 26.29 | 50 |
| (Disease management or Case management).mp. and (care or health or healthcare).mp. and og.xs. | 46 | 21.6 | 51 |
| (Disease management or Case management).mp. and (care or health or healthcare).mp. and (og.xs. or services.mp.) | 50 | 23.47 | 82 |
| (Disease management or Case management).mp. and (care or health or healthcare).mp. and (og.xs. or services.mp. or delivery.mp.) | 52 | 24.41 | 76 |
| (Disease management or Case management).mp. and (care or health or healthcare).mp. and (og.xs. or services.mp. or delivery.mp. or systems.mp.) | 52 | 24.41 | 73 |
| (Disease management or Case management).mp. and (care or health or healthcare).mp. and (og.xs. or services.mp. or delivery.mp. or model.mp.) | 53 | 24.88 | 74 |
| (Disease management or Case management).mp. and (care or health or healthcare).mp. and (og.xs. or services.mp. or delivery.mp. or model.mp. or organi?ational.mp.) | 53 | 24.88 | 70 |
| (Disease management or Case management).mp. and (care or health or healthcare).mp. and (og.xs. or services.mp. or delivery.mp. or model.mp. or quality.mp.) | 55 | 25.82 | 69 |
| (Disease management or Case management).mp. and (care or health or healthcare).mp. and (og.xs. or services.mp. or delivery.mp. or model.mp. or quality.mp. or service.mp.) | 55 | 25.82 | 69 |
| (Disease management or Case management).mp. and (care or health or healthcare).mp. and (og.xs. or services.mp. or delivery.mp. or model.mp. or quality.mp. or models.mp.) | 55 | 25.82 | 70 |
| **AND’d search** | | | |
| (Disease management or Case management).mp. and (care AND (health OR healthcare)).mp. | 52 | 24.42 | 67 |
| (Disease management or Case management).mp. and (care AND (health OR healthcare)).mp. and og.xs | 46 | 21.6 | 56 |
| (Disease management or Case management).mp. and (care and (health or healthcare)).mp. and (og.xs. or services.mp.) | 48 | 22.54 | 60 |
| (Disease management or Case management).mp. and (care and (health or healthcare)).mp. and (og.xs. or services.mp. or delivery.mp) | 50 | 23.47 | 68 |
| ((Disease management or Case management) and (care and (health or healthcare))).mp. and (og.xs. or services.mp. or delivery.mp. or systems.mp.) | 50 | 23.47 | 76 |
| ((Disease management or Case management) and (care and (health or healthcare))).mp. and (og.xs. or services.mp. or delivery.mp. or model.mp.) | 50 | 23.47 | 75 |
| ((Disease management or Case management) and (care and (health or healthcare))).mp. and (og.xs. or services.mp. or delivery.mp. or organi?ational.mp.) | 50 | 23.47 | 72 |
| ((Disease management or Case management) and (care and (health or healthcare))).mp. and (og.xs. or services.mp. or delivery.mp. or quality.mp.) | 52 | 24.41 | 73 |
| ((Disease management or Case management) and (care and (health or healthcare))).mp. and (og.xs. or services.mp. or delivery.mp. or quality.mp. or service.mp.) | 52 | 24.41 | 73 |
| ((Disease management or Case management) and (care and (health or healthcare))).mp. and (og.xs. or services.mp. or delivery.mp. or quality.mp. or models.mp.) | 52 | 24.41 | 73 |

**Best OR combination** (with minimum number of terms)
Recall: 25.82%; Precision: 69%

(Disease management or Case management).mp. and (care **or** health or healthcare).mp. and (og.xs. or services.mp. or delivery.mp. or model.mp. or quality.mp.)

**Best AND combination** (with minimum number of terms)
Recall: 24.41%; Precision: 73%

((Disease management or Case management) and (care **and** (health or healthcare))).mp. and (og.xs. or services.mp. or delivery.mp. or quality.mp.)

To determine the most effective search in retrieving the balance of the FDS once search component one was conducted (i.e n=60), we combined each with the balance using AND. The OR’d search retrieved n=27 of the 60 and the AND’d search retrieved only 24 citations.

The final disease management component (component two) was therefore the OR version:

(Disease management or Case management).mp. and (care **or** health or healthcare).mp. and (og.xs. or services.mp. or delivery.mp. or model.mp. or quality.mp.)

Combining component two with component one, the search becomes:

(((Integrat* or coordinat*) and care and (health or healthcare)).mp. and (og.xs. or services.mp. or delivery.mp. or management.mp. or systems.mp. or model.mp. or organi?ational.mp. or quality.mp.)) OR (((Disease management or Case management) and (care or health or healthcare)).mp. and (og.xs. or services.mp. or delivery.mp. or model.mp. or quality.mp.))

FDS retrieval increases to 180/213 = 84.51%
